# Pramef12 enhances reprogramming into naïve iPS cells

**DOI:** 10.1016/j.bbrep.2022.101267

**Published:** 2022-05-10

**Authors:** Daiki Haraguchi, Toshinobu Nakamura

**Affiliations:** a Gaduate School of Bio-Science, Japan; b Department of Bio-Science, Japan; c Genome Editing Research Institute, Ngahama Institute of Bio-Science and Technology, Shiga, 526-0829, Japan

**Keywords:** iPS cells, Reprogramming, Pramef12, Wnt/β-catenin pathway

## Abstract

Somatic cells can be reprogrammed into induced pluripotent stem (iPS) cells by forced expression of the transcription factors Oct3/4, Klf4, Sox2, and c-Myc (OKSM). Somatic cell nuclear transfer can also be utilized to reprogram somatic cells into totipotent embryos, suggesting that factors present in oocytes potentially enhance the efficiency of iPS cell generation. Here, we showed that preferentially expressed antigen of melanoma family member 12 (Pramef12), which is highly expressed in oocytes, enhances the generation of iPS cells from mouse fibroblasts. Overexpression of Pramef12 during the early phase of OKSM-induced reprogramming enhanced the efficiency of iPS cell derivation. In addition, overexpression of Pramef12 also enhanced expression of naïve pluripotency-associated genes, Gtl2 located within the *Dlk1–Dio3* imprinted region essential for full pluripotency, glycolysis-associated genes, and oxidative phosphorylation-associated genes, and it promoted mesenchymal-to-epithelial transition during iPS cell generation. Furthermore, Pramef12 greatly activated β-catenin during iPS cell generation. These observations suggested that Pramef12 enhances OKSM-induced reprogramming via activation of the Wnt/β-catenin pathway.

## Highlights

• Pramef12 enhances OKSM-induced reprogramming into naïve iPS cells.
• Pramef12 enhances expression of naïve pluripotency-associated genes, essential genes for full pluripotency, glycolysis-associated genes, and oxidative phosphorylation-associated genes.
• Pramef12 promotes mesenchymal-to-epithelial transition during iPS cell generation.
• Pramef12 enhances OKSM-induced reprogramming via activation of the Wnt/β-catenin pathway.

## 1 Introduction

Induced pluripotent stem (iPS) cells can be generated from somatic cells by ectopic expression of the transcription factors Oct3/4, Klf4, Sox2, and c-Myc (OKSM). iPS cells closely resemble embryonic stem (ES) cells, which can differentiate into every somatic cell type of the body and possess the capacity of unlimited replication [1]. As use of iPS cells is not associated with the ethical concerns related to ES cell derivation and issues with potential allogeneic immune rejection, iPS cells are ideal for producing patient- and disease-specific cells for future clinical applications, regenerative medicine, and drug development [2,3].

Mammalian oocytes have the ability to reprogram somatic cells into totipotent embryos [4,5]. Importantly, nuclear transfer ES cells derived from somatic cell nuclear transfer embryos exhibit gene expression and DNA methylation patterns more similar to those of ES cells than iPS cells [6]. In addition, OKSM are expressed at high levels in ES cells but low levels in oocytes, suggesting that oocytes contain other reprogramming factor(s) [7]. As only four factors are used in OKSM-induced reprogramming, versus the large number of oocyte-derived factors used in somatic cell nuclear transfer-induced reprogramming, it is likely that oocyte-derived factor(s) can improve the generation efficiency and quality of iPS cells. Indeed, oocyte-derived factors, such as Glis1 [8], histone variants, TH2A and TH2B [9], Dppa3 (also known as Stella or PGC7) [10], Oobox1 [11], Zscan4c [12], and Zscan4f [13], can improve the generati on efficiency and/or quality of iPS cells.

As the genes important for somatic cell nuclear reprogramming are likely to be expressed specifically in totipotent cells, we screened for genes specific to totipotent early pre-implantation embryos. We found that preferentially expressed antigen of melanoma family member 12 (Pramef12) was expressed specifically in early pre-implantation embryos. Here, we examine the effects of Pramef12 on OKSM-induced reprogramming and found that overexpression of Pramef12 during the early phase of reprogramming enhanced the efficiency of iPS cell generation. Prmaef12 also promoted the expression of naïve pluripotency-associated genes, metabolic transition, mesenchymal-to-epithelial transition (MET), and activation of Gtl2 located within the imprinted *Dlk1–Dio3* cluster. Finally, we showed that overexpression of Pramef12 during OKSM-induced reprogramming markedly activated the Wnt/β-catenin pathway.

## 2 Materials and methods

### 2.1 Cell cultures

MEFs were isolated from embryonic day 13.5 (E13.5) embryos of Jcl:ICR or Pramef12-knockout mice (Shinchi A and Nakamura T, in preparation) and cultured in Dulbecco's modified Eagle's medium (DMEM; Nacalai Tesque) supplemented with 10% FCS. Feeder-free mouse ES-E14tg2a and iPS cells were maintained as described previously [14].

### 2.2 Plasmid construction

Pramef12 cDNA was amplified by PCR and cloned into PB-TRE3G-cHApA [15] to yield PB-TRE3G-Pramef12. OKS and c-Myc were amplified by PCR from PB-TRE3G-OKS [15] and PB-TRE3G-c-Myc [15], respectively, and cloned into PB-CAG to yield PB-CAG-OKS and PB-CAG-c-Myc, respectively.

### 2.3 iPS generation

MEFs were plated at 1 × 10^5^ cells per well in 6-well plates and incubated overnight. MEFs were simultaneously transfected with 1 μg PB-CAG-OKS, 1 μg PB-CAG-c-Myc, 1 μg of PB-TRE3G-Pramef12, 1 μg PB-CAG-rtTA, and 1 μg CAG-HyPBase using Xfec Transfection Reagent (Takara) according to the manufacturer's instructions. One day after transfection, the medium was replaced with GMEM (Sigma) supplemented with 10% FCS, 1 mM sodium pyruvate (Wako), 1 × MEM non-essential amino acids (Wako), 0.1 mM 2-mercaptoethanol (2-ME; Nacalai Tesque), 1000 U/ml LIF, and 1 μg/ml Dox (Sigma). Five days after transfection, 1 × 10^5^ cells were reseeded on mitomycin-treated MEF feeder layers. After an additional 10 days in culture, ES-like colonies were picked, dissociated, and plated on fresh mitomycin-treated MEF feeder layers for analysis.

### 2.4 Alkaline phosphatase (ALP) staining

ALP staining was performed using the ALP staining kit (Stemgent) according to the manufacture's instruction.

### 2.5 Immunostaining

Immunostaining was performed as previously reported [16]. iPS cells were fixed with 4% paraformaldehyde in phosphate-buffered saline (PBS), permeabilized with 0.2% Triton X-100 in PBS for 5min and blocked in 5% normal goat serum in PBS. Anti-Nanog (1:500, 14-5761-80; eBioscience) antibody were applied to the cells and incubated for 1 h. After washing with PBS, the cells were incubated with goat anti-rat IgG Alexa 568 (1:500, A-11077; Thermo Fisher Science) and DAPI (1 μg/mL, 40–07971; Dojindo). Immunofluorescence were observed using Fluorescence Microscope BZ-X700 (KEYENCE) and Nanog positive cells were determined using Image J.

### 2.6 RNA extraction and quantitative real time RT-PCR (RT-qPCR)

Total RNA was isolated using the RNeasy mini kit (Qiagen), and 300 ng total RNA was used for cDNA synthesis. Reverse transcription was performed using the SuperScript VILO cDNA Synthesis Kit (Invitrogen) according to the manufacturer's instructions. Real-time PCR was performed using the LightCycler 96 System (Roche) with the KAPA SYBR FAST qPCR Kit (Kapa Biosystems). Transcript levels were determined in triplicate reactions and normalized to β-actin. The primers used in this study are shown in Table S1.

### 2.7 Western blot analysis

Western blot analysis was performed as described previously. The primary antibodies used were anti-β-catenin (clone14/Beta-Catenin, 1:200, #610154; BD Transduction Laboratories), anti-active β-catenin (clone 8E7, 1:200, #06–665; Millipore), and anti-β-actin (1:10,000, AC-15; Sigma). Band intensities were measured using Image J, and normalized to β-actin.

## 3 Results and discussion

### 3.1 Pramef12 expression in oocyte and preimplantation embryos

To screen for totipotent cell-specific genes, digital differential display analysis was performed to compare the mouse expressed sequence tag libraries among 21 totipotent cell libraries (oocyte, zygote, and 2-, 4-, 8-, and 16-cell embryos) and another 704 pooled libraries including pluripotent ES cells, blastocysts, and various embryonic and adult tissues. We identified Pramef12, a member of the preferentially expressed antigen of melanoma (PRAME) multigene family, as a candidate totipotent cell-specific gene. PRAME genes were first discovered in human melanoma cell lines and are tumor-associated antigens recognized by cytolytic T lymphocytes. Real-time quantitative reverse transcription PCR (RT-qPCR) analysis showed that Pramef12 mRNA was expressed in GV oocytes, MII oocytes, zygotes, and 2-cell embryos, but not in any adult tissues (Supplementary Fig. S1A). To confirm these results, we extracted RNA sequencing data for Pramef12 from the Database of Transcriptome in Mouse Early Embryos (DBTMEE) and found that Pramef12 mRNA was expressed in oocytes, zygotes, and 2- and 4-cell embryos (Supplementary Fig. S1B).

### 3.2 Pramef12 enhances iPS cell generation

**Fig. 1:**
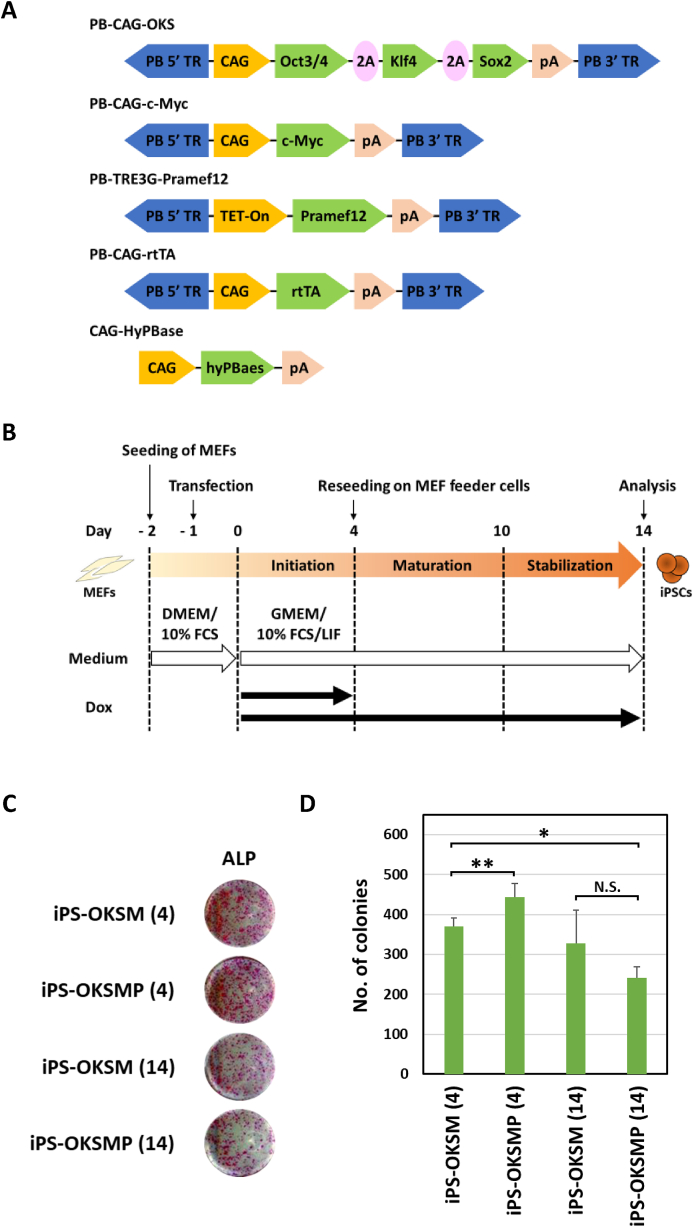
Overexpression of Pramef12 enhances iPS cell generation (A) Construction of the vectors used in this study. (B) Scheme of the experimental design showing generation of iPS cells. (C) iPS colonies generated using OKSM or OKSMP were stained for ALP 14 days after reprogramming. (D) Numbers of APL-positive colonies generated using OKSM or OKSMP 14 days after reprogramming. The numbers in parentheses indicate the number for days for which Dox was added. Data are presented as the mean ± s.d. (*n* = 3). *P < 0.05, **P < 0.01 (Student's *t*-test).

Recently, it was reported that some oocyte-derived factors can enhance the reprogramming efficiency and quality of iPS cells [[8], [9], [10], [11], [12], [13]]. We therefore examine whether Pramef12 enhances the efficiency of OKSM-mediated reprogramming and quality of iPS cells. As Pramef12 was not expressed in ES/iPS cells, we examined the efficiency of iPS cell derivation when Pramef12 was expressed during the early phase of the reprogramming process or continuously expressed during the reprogramming process. For this purpose, we used PB-CAG-OKS, PB-CAG-c-Myc, PB-TRE3G-Pramef12, PB-CAG-rtTA, and CAG-HyPBase vectors to induce iPS cells from somatic cells (Fig. 1A). First, we examined whether Pramef12 expression could be regulated by the TetON system we used. We found that Pramef12 was expressed 4 h after the addition of Dox and maintained its expression until 24 h (Supplementary Fig. S2). We also found that Pramef12 expression was revearted to a basal level within 36 h after Dox withdraw (Supplementary Fig. S2). Mouse embryonic fibroblasts (MEFs) were transfected with all of these vectors simultaneously and cultured in Glasgow minimum essential medium (GMEM) supplemented with 10% fetal calf serum (FCS) and leukemia inhibitory factor (LIF). Four days after transfection, the MEFs were reseeded onto a mitomycin C-treated MEF feeder layer and cultured in GMEM with 10% FCS and LIF for 10 days (Fig. 1B). To induce Pramef12 expression, doxycycline (Dox) was added to the culture medium for the initial 4 and 14 days during the iPS cell generation process (iPS-OKSMP (4) and iPS-OKSMP (14), respectively) (Fig. 1B). We found that expression of Pramef12 during the early phase of OKSM-induced reprogramming enhanced the number of alkaline phosphatase (ALP)-positive colonies, indicating that overexpression of Pramef12 increased the efficiency of iPS cell derivation (Fig. 1C and D). In contrast, expression of Pramef12 entire OKSM-induced reprogramming process significantly reduced the number of ALP-positive colonies, suggesting that Pramef12 acts on OKSM-induced reprogramming in cell context-dependent manner. These results suggest that Pramef12 plays an important role in the early phase of OKSM-induced reprogramming.

### 3.3 Pramef12 can facilitate naïve iPS cell generation

**Fig. 2:**
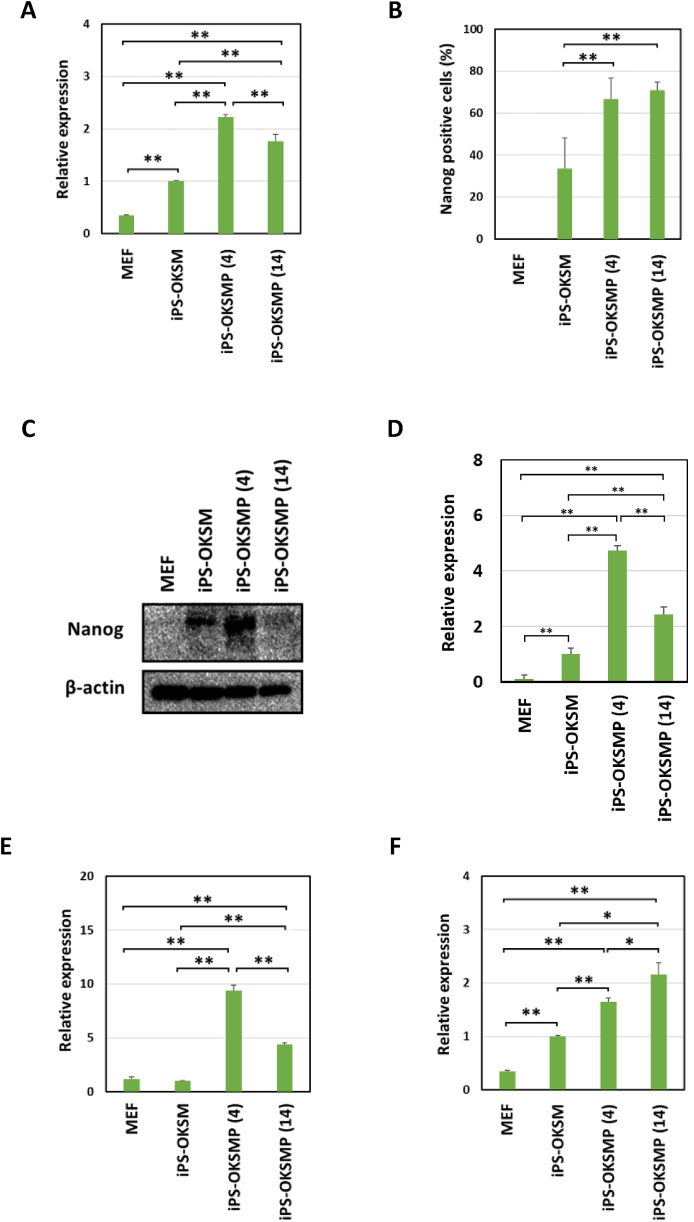
Pramef12 can induce high-quality iPS cells (A) Relative levels of *Nanog* mRNA in MEF and iPS cells generated using OKSM or OKSMP. (B) Percentages of Nanog-positive cells in MEF and iPS cells generated using OKSM or OKSMP determined by immunostaining. (C) Nanog protein levels in MEF and iPS cells generated using OKSM or OKSMP analyzed by Western blotting. (D) The level of Nanog protein determined by measuring the intensities of the bands shown in (C). (E) Relative levels of *Dppa4* mRNA in MEF and iPS cells generated using OKSM or OKSMP. (F) Relative expression level of *Gtl2* in MEF and iPS cells generated using OKSM or OKSMP. Data are presented as the mean ± s.d. (*n* = 3). *P < 0.05, **P < 0.01 (Student's *t*-test). Representative results of one iPS clone were shown, and similar results were obtained at least three independent iPS clones. The numbers in parentheses indicate the number of days the cells were incubated with Dox.

To characterize iPS cells generated using Pramef12 in addition to OKSM (iPS-OKSMP), we examined the expression of *Nanog* mRNA. As shown in Fig. 2A, the level of Nanog expression was significantly higher in iPS-OKSMP (4) and iPS-OKSMP (14) than in iPS-OKSM. The percentage of Nanog-positive cells was significantly increased in iPS-OKSMP (4) and iPS-OKSMP (14) compared with iPS-OKSM (Fig. 2B). Furthermore, the level of Nanog protein expression was significantly higher in iPS-OKSMP (4) and iPS-OKSMP (14) than in iPS-OKSM (Fig. 2C and D). We next examined the expression of naïve pluripotency-associated gene Dppa4. The levels of Dppa4 expression was significantly higher in iPS-OKSMP (4) and iPS-OKSMP (14) than in iPS-OKSM (Fig. 2E). We found that the level of *Gtl2* RNA expression were significantly higher in iPS-OKSMP (4) and iPS-OKSMP (14) than in iPS-OKSM (Fig. 2F). As reported previously, Nanog-high ES cells possess high self-renewal efficiency, whereas Nanog-low ES cells show an increased propensity for extraembryonic ectodermal differentiation, and therefore Nanog acts to stabilize pluripotency [[17], [18], [19]]. It was reported that low-quality chimera-forming iPS cells exhibit aberrant silencing of coding and noncoding genes located in the *Dlk1–Dio3* imprinting cluster [20,21]. It has been also reported that activation of the imprinted *Dlk1–Dio3* region was shown to be necessary for acquisition of full pluripotency in OKSM-induced iPS cells [20,21]. As overexpression of Pramef12 during the early phase of OKSM-induced reprogramming enhanced the expression of Nanog, Dppa4, and Gtl2, Pramef12 promoted OKSM-induced reprogramming into high-quality naïve pluripotency.

### 3.4 Gene expression change induced by Pramef12 during reprogramming

To determine the role of endogenous Pramef12, we performed OKSM-induced reprogramming of homozygous Pramef12−/− knockout MEFs. iPS cells were generated even in the absence of Pramef12, indicating that endogenous Pramef12 does not contribute to OKSM-induced reprogramming (Supplementary Fig. S3). In addition, expression of Pramef12 during the early phase of OKSM-induced reprogramming significantly enhanced the efficiency of iPS cell generation, as in the case of wild-type MEFs (Supplementary Fig. S3).

**Fig. 3:**
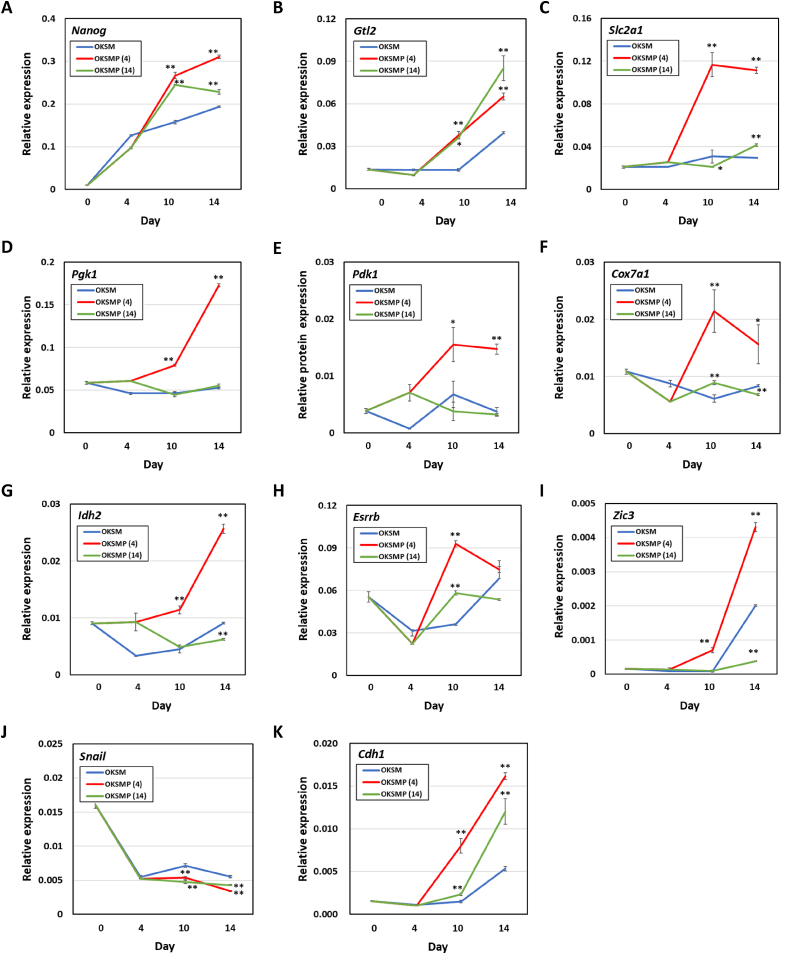
Pramef12 enhances somatic cell reprogramming by acting on middle phase. Time course analysis of the expression of *Nanog* (A), *Gtl2* (B), *Slc2a1* (C), *Pgk1* (D), *Pdk1* (E), *Cox7a1* (F), *Idh2* (G), *Esrrb* (H), *Zic3* (I), *Snail* (J), and *Cdh1* (K) determined by RT-qPCR. Data are presented as the mean ± s.d. (*n* = 3). *P < 0.05, **P < 0.01 (Student's *t*-test). The numbers in parentheses indicate the number of days the cells were incubated with Dox.

To determine how Pramef12 promotes OKSM-induced reprograming, we analyzed the expression of a subset of key reprogramming-associated genes, including pluripotency-associated genes (*Nanog* and *Esrrb*), imprinted gene (*Gtl2*), glycolysis-associated genes (*Slc2a1*, *Pgk1*, and *Pdk1*), an oxidative phosphorylation-associated gene (*Cox7a1* and *Idh2*), energy metabolism regulatory genes *(Esrrb* and *Zic3*), and epithelial-to-mesenchymal transition (EMT)-associated genes (*Snail* and *Chd1*). Overexpression of Pramef12 during OKSM-induced reprogramming significantly upregulated the expression of *Nanog*, *Gtl2*, and *Esrrb* on day 10 regardless of the duration of Pramef12 expression (Fig. 3A, B, H). The expression levels of *Slc2a1*, *Pgk1*, and *Pdk1 Cox7a1* and *Idh2 Zic3*were significantly upregulated in cells undergoing OKSMP (4)-induced, but not OKSMP (14)-induced, reprogramming on day 10 (Fig. 3C–G, I). Expression of Snail was slightly downregulated in cells undergoing OKSMP (4)- and OKSMP (14)-induced reprogramming on day 10 regardless of the duration of Pramef12 expression (Fig. 3J). Cdh1 (also known as E-cadherin), was upregulated in cells undergoing OKSMP (4)-induced, but not OKSMP (14)-induced, reprogramming on days 10 and 14 (Fig. 3K). Reprogramming of human or mouse somatic cells to iPS cells requires a shift from mainly OXPHOS to mainly glycolytic metabolism [12,22,23]. However, transient activation of OXPHOS was shown to be required for OKSM-induced reprogramming [24]. In addition, it has been reported that naïve pluripotent stem cells utilize both glycolytic and OXPHOS pathways, whereas primed pluripotent stem cells exclusively utilize glycolysis for energy production [[25], [26], [27], [28]]. A recent study showed that two transcription factors, Zic3 and Esrrb, synergistically promote induction of naïve pluripotency during OKSM-induced reprogramming [29]. In this study, we demonstrated that overexpression of Pramef12 during the early phase of OKSM-induced reprogramming upregulated the expression of both glycolysis- and OXPHOS-associated genes. In addition, overexpression of Pramef12 also increased the expression of Zic3 and Esrrb during OKSM-induced reprogramming, suggesting that Pramef12 may activate both glycolytic and OXPHOS pathways for energy production via upregulated expression of these two transcription factors. It has been reported that derivation of iPS cells from somatic cells requires MET by suppressing EMT signals, including Snail expression, and activating epithelial programming, such as Cdh1 upregulation [30,31]. Overexpression of Pramef12 during OKSM-induced reprogramming significantly increased the expression of Cdh1 and slightly reduced the expression of Snail, suggesting that Pramef12 may enhance activation of epithelial programming rather than suppressing EMT signals.

### 3.5 Pramef12 activate Wnt/β-catenin pathway during reprogramming

**Fig. 4:**
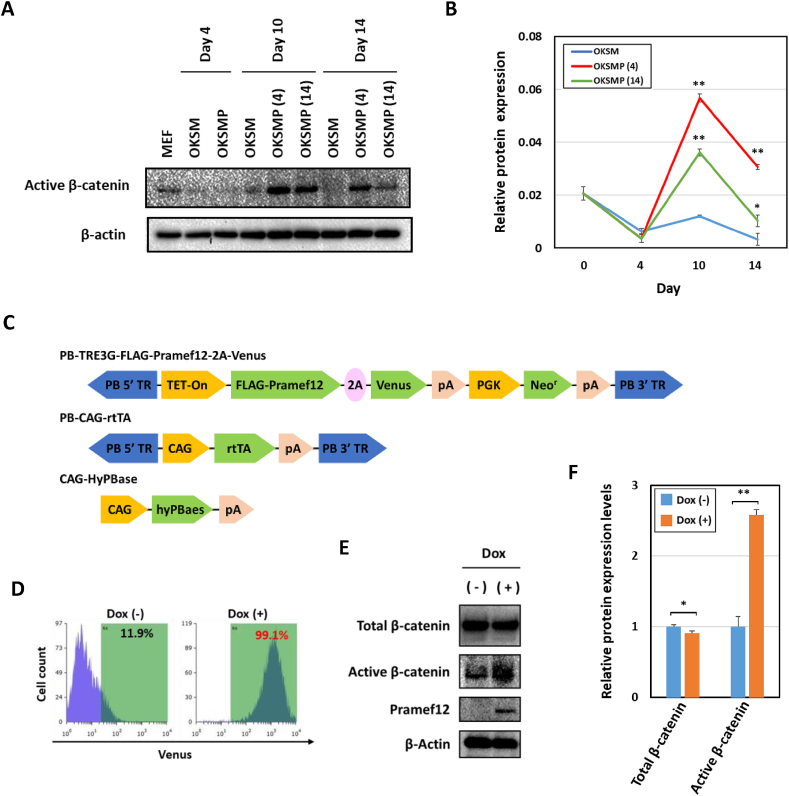
Pramef12 activates β-catenin during OKSM-induced reprogramming (A) Time course analysis of active β-catenin protein levels. (B) Active β-catenin protein levels determined by measuring the intensities of the bands shown in (A). Data are presented as the mean ± s.d. (*n* = 3). *P < 0.05, **P < 0.01 (Student's *t*-test). The numbers in parentheses indicate the number of days the cells were incubated with Dox. (C) Construction of the vectors used to generate ES cells harboring inducible Pramef12. (D) FACS analysis of ES cells with inducible Pramef12 in the presence or absence of Dox. (E) Protein levels of total β-catenin, active β-catenin, Pramef12, and β-actin in ES cells with inducible Pramef12 in the presence or absence of Dox. (F) The expression levels of total and active β-catenin proteins determined by measuring the intensities of the bands shown in (E). Data are presented as the mean ± s.d. (*n* = 3). *P < 0.05, **P < 0.01 (Student's *t*-test).

Wnt3a and glycogen synthase kinase 3 inhibitors have been shown to successfully simulate both OKSM- and cell fusion-induced reprogramming via activation of β-catenin. These reports prompted us to examine whether Pramef12 affects the Wnt/β-catenin pathway [[32], [33], [34]]. The β-catenin activity was analyzed at successive time points during reprogramming. Western blot analysis showed that β-catenin was markedly activated in OKSMP (4)- and OKSMP (14)-induced cells, but not OKSM-induced cells (Fig. 4A and B). In addition, ectopic expression of Pramef12 induced activation of β-catenin in ES cells (Fig. 4C–F). These results suggest that Pramef12 enhanced OKSM-induced reprogramming at least partially via the Wnt/β-catenin pathway. Wnt3a and GSK-3 inhibitors have been shown to enhance both cell fusion-induced and OKSM-induced reprograming [[32], [33], [34]]. It has been reported that enhancement of OKSM-induced reprogramming by Wnt/β-catenin signaling is due to upregulated expression of pluripotency circuitry genes and is not due to an increase in the total cell population or activation of c-Myc [34]. The Wnt/β-catenin pathway regulates multiple cellular processes, including proliferation, differentiation, cell fate, and organogenesis [35]. A recent study showed that Pdk1 is a downstream gene of the Wnt/β-catenin pathway that promotes glycolysis by inhibiting pyruvate flux into mitochondrial respiration in colon cancer [36]. Another study showed that the Wnt/β-catenin pathway positively regulated the expression of coding and noncoding genes located in the *Dlk1–Dio3* imprinting cluster during mouse liver tumor promotion [21]. It has been reported that Esrrb is a central downstream factor of the Wnt/β-catenin pathway that regulates tissue-scale organization and maintenance of the mouse pluripotent lineage [37]. Thus, activation of glycolysis-associated genes, *Dlk1–Dio3* imprinting cluster genes, and Esrrb during OKSM-induced reprogramming by overexpression of Pramef12 is mediated at least partially by the Wnt/β-catenin pathway. In this study, we showed that Pramef12 was continuously localized in the cytoplasm during OKSM-induced reprogramming (Supplementary Fig. S4). Although the molecular mechanism by which Pramef12 activates the Wnt/β-catenin pathway remains to be elucidated, considering its cytoplasmic localization, it is possible that Pramef12 inhibits the β-catenin destruction complex. Further studies will enable high-quality iPS generation as well as valuable insights into the molecular mechanisms of OKSM-induced reprogramming.

## Declaration of competing interest

The authors declare that they have no known competing financial interests or personal relationships that could have appeared to influence the work reported in this paper.

## Data Availability

No data was used for the research described in the article.
