# Supplementary material for: Pramef12 enhances reprogramming into naïve iPS cells
Source: Biochem Biophys Rep. 2022 May 10;30:101267. doi: 10.1016/j.bbrep.2022.101267 (PMC9111934; doi:10.1016/j.bbrep.2022.101267)
Supplement: Multimedia component 1 [file mmc1.pptx]

## Slide 1
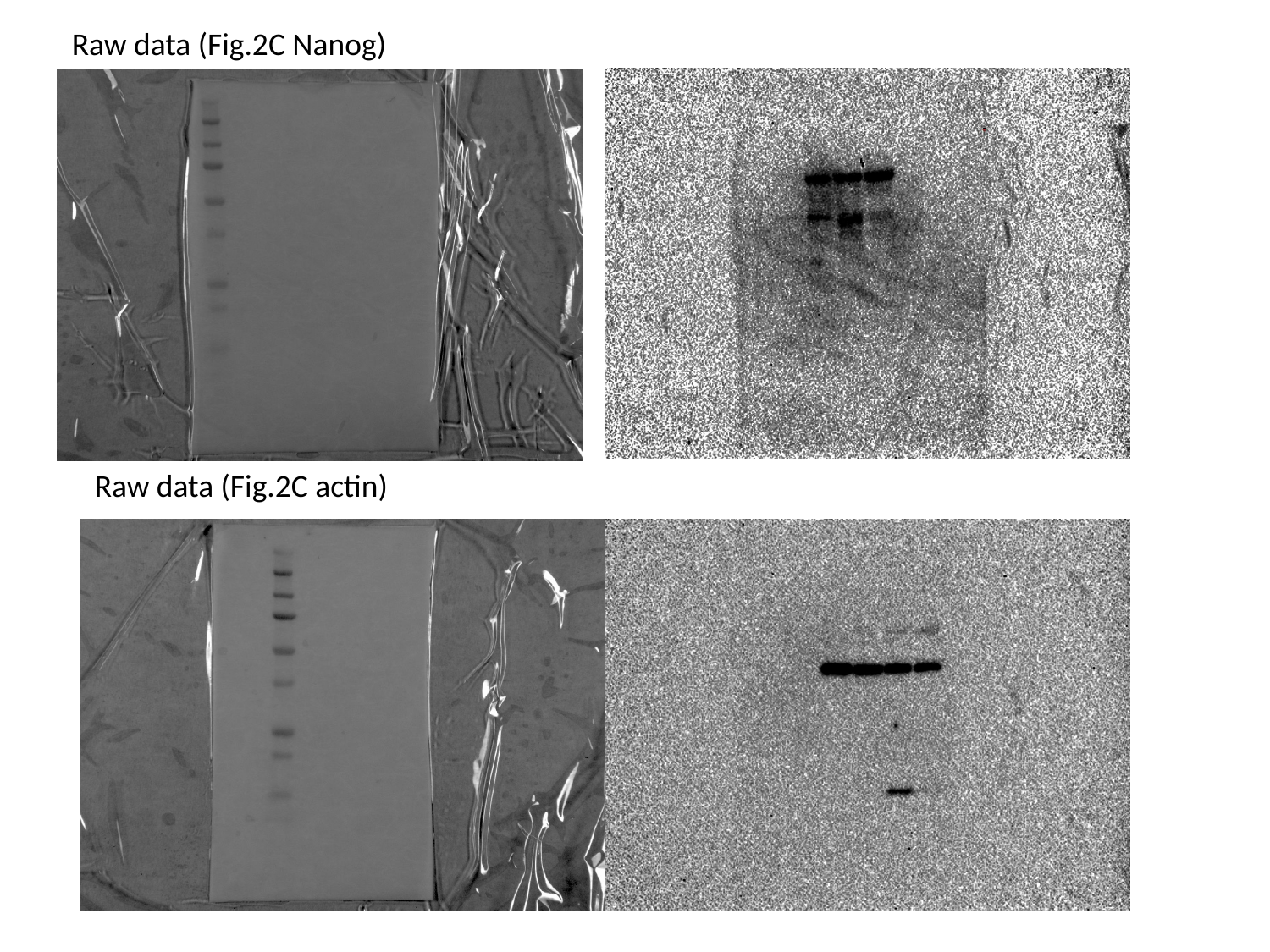

Raw data (Fig.2C Nanog)
Raw data (Fig.2C actin)

## Slide 2
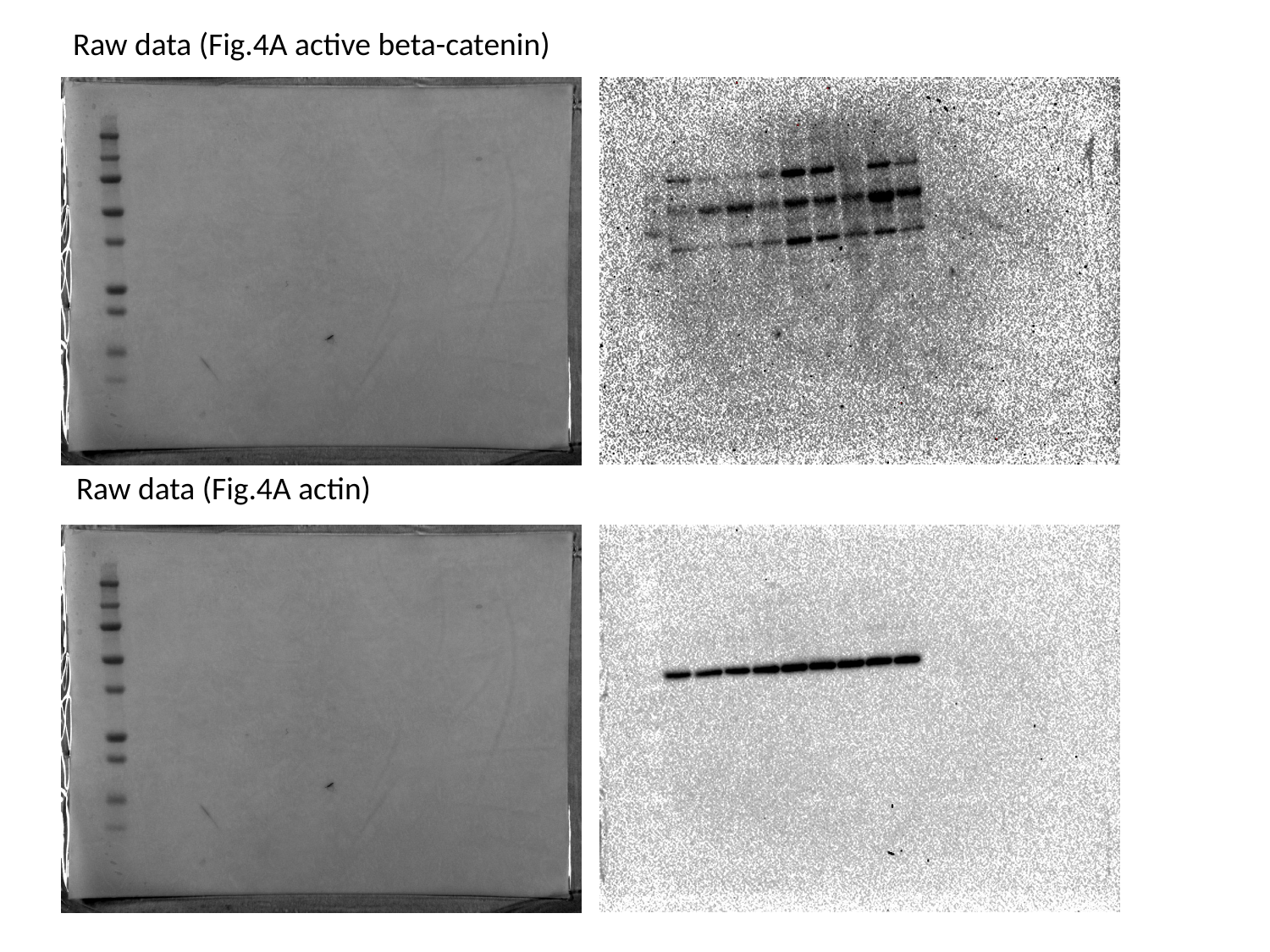

Raw data (Fig.4A active beta-catenin)
Raw data (Fig.4A actin)

## Slide 3
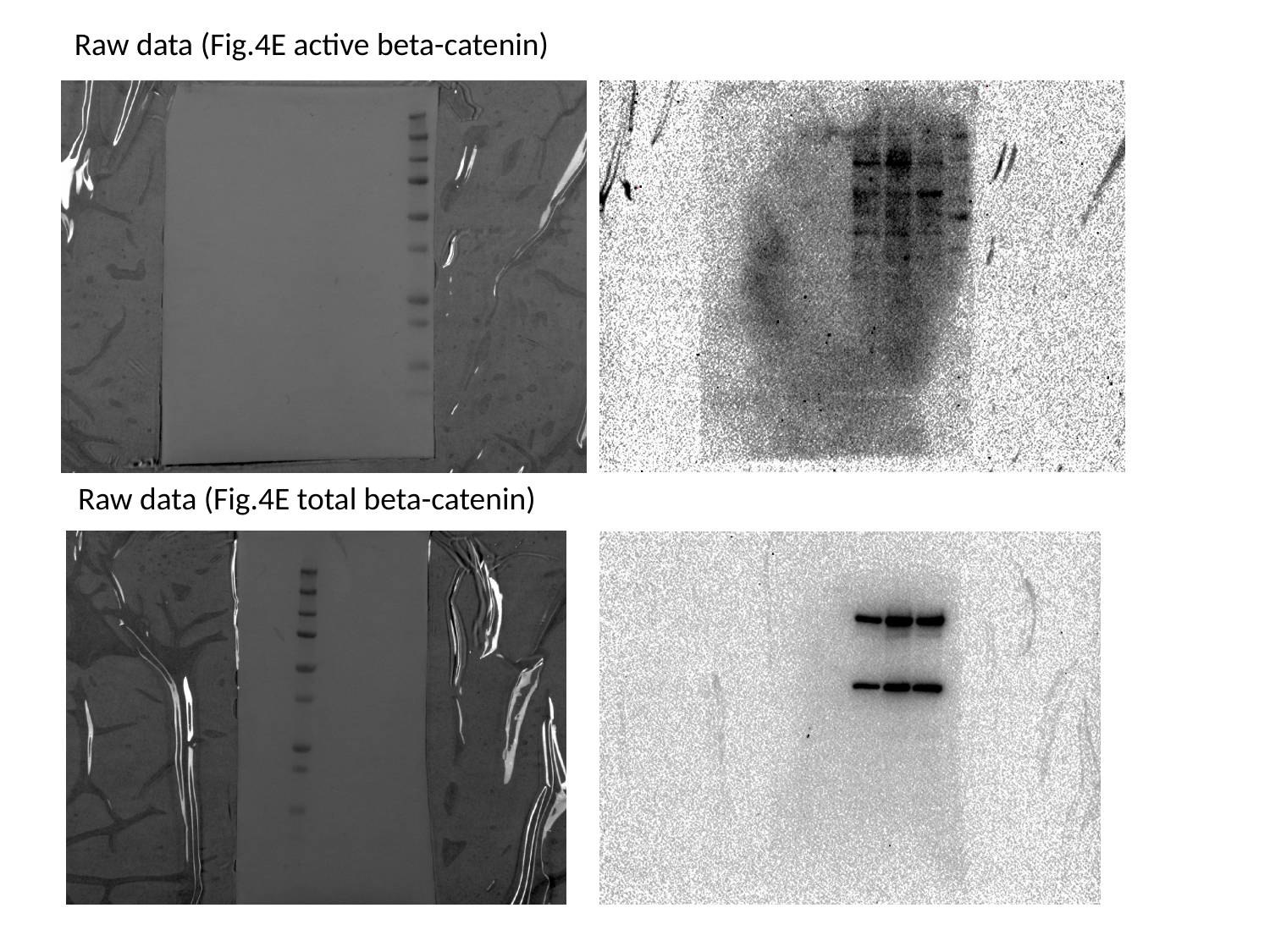

Raw data (Fig.4E active beta-catenin)
Raw data (Fig.4E total beta-catenin)

## Slide 4
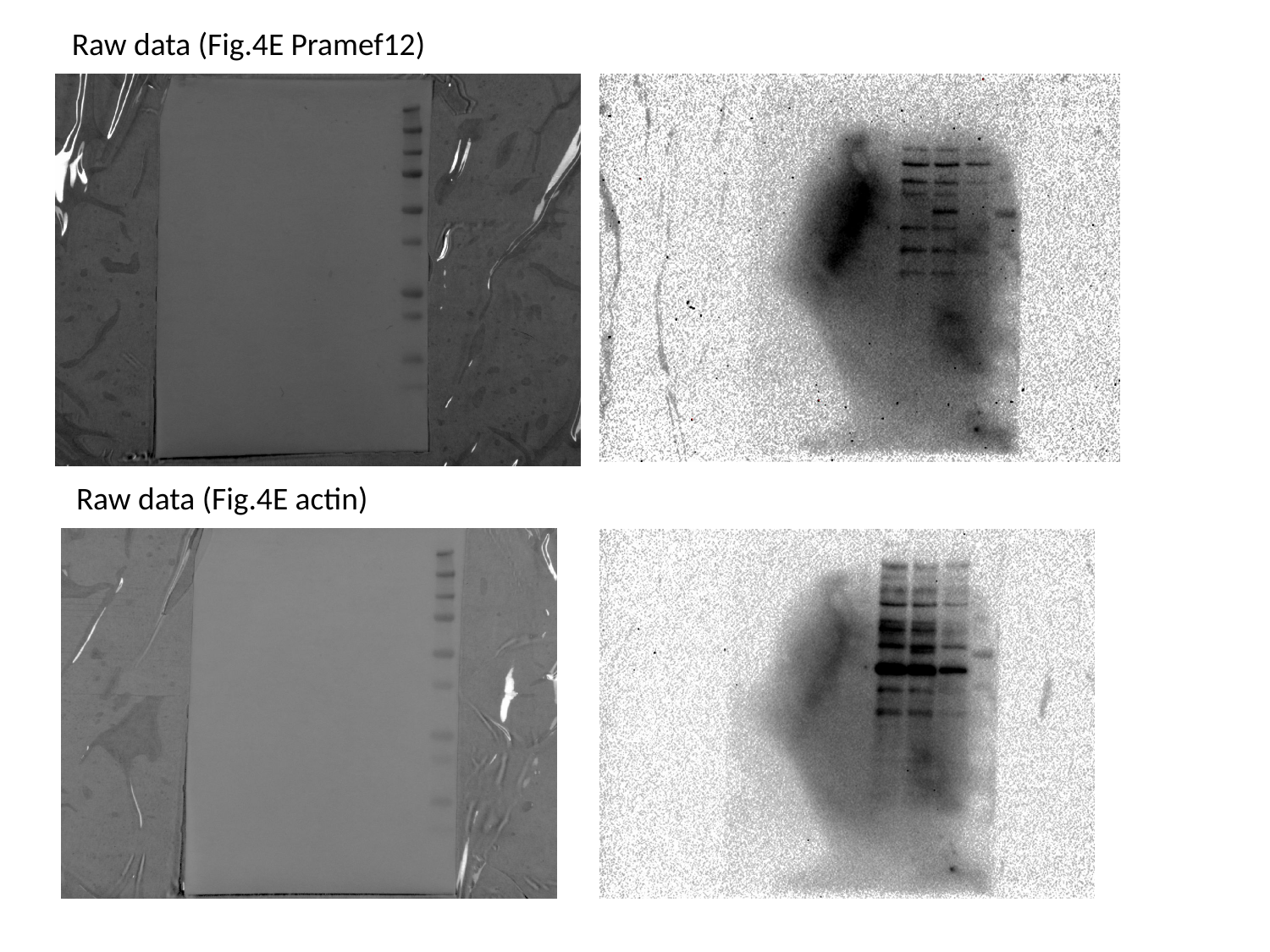

Raw data (Fig.4E Pramef12)
Raw data (Fig.4E actin)
