# Supplementary material for: Pramef12 enhances reprogramming into naïve iPS cells
Source: Biochem Biophys Rep. 2022 May 10;30:101267. doi: 10.1016/j.bbrep.2022.101267 (PMC9111934; doi:10.1016/j.bbrep.2022.101267)
Supplement: Multimedia component 2 [file mmc2.doc]

**Table S1** Primer sequences

| Name | Sequence (5’ to 3’) |
| --- | --- |
| pramef12_ORF_FWD | ATGAGCTTGCGTGCCACACC |
| pramef12_ORF_REV | GGAGCTCCATGGGCAGGTCC |
| Nanog (FWD) | CACCCACCCATGCTAGTCTT |
| Nanog (REV) | ACCCTCAAACTCCTGGTCCT |
| Dppa4_FWD | AACCAAGCACGGCTCCTGCAAA |
| Dppa4_REV | ACCTTCCTACGAGTCTGTCCTG |
| Gtl2_qPCR_FWD2 | AGAGAGACCCACCTACTG |
| Gtl2_qPCR_REV2 | CTGTGAGGTAGGAACCTG |
| Slc2a1_qPCR_FWD | TCCCAGCAGCAAGAAGGTG |
| Slc2a1_qPCR_REV | GCGGTGGTTCCATGTTTGAT |
| Pgk1-S | CAGCCTTGATCCTTTGGTTG |
| Pgk1-AS | CTGACTTTGGACAAGCTGGA |
| Pdk1_qPCR_FWD | GTTGAAACGTCCCGTGCT |
| Pdk1_qPCR_REV | GCGTGATATGGGCAATCC |
| Cox7a1_qPCR_FWD | CGAAGAGGGGAGGTGACTC |
| Cox7a1_qPCR_REV | AGCCTGGGAGACCCGTAG |
| Idh2_qPCR_FWD | GGATGTACAACACCGACGAGT |
| Idh2_qPCR_REV | CGGCCATTTCTTCTGGATAG |
| Esrrb (FWD) | TTTCTGGAACCCATGGAGAG |
| Esrrb (REV) | AGCCAGCACCTCCTTCTACA |
| Zic3_qPCR_FWD2 | TCCTTCAAGGCGAAGTACAAACTG |
| Zic3_qPCR_REV2 | GGTTTCTCACCTGTATGGGTCCT |
| Snail_FWD | TGTCTGCACGACCTGTGGAAAG |
| Snail_REV | CTTCACATCCGAGTGGGTTTGG |
| E-cadherin_FWD | GGTCATCAGTGTGCTCACCTCT |
| E-cadherin_REV | GCTGTTGTGCTCAAGCCTTCAC |
