# Supplementary material for: Pramef12 enhances reprogramming into naïve iPS cells
Source: Biochem Biophys Rep. 2022 May 10;30:101267. doi: 10.1016/j.bbrep.2022.101267 (PMC9111934; doi:10.1016/j.bbrep.2022.101267)
Supplement: Multimedia component 3 [file mmc3.pptx]

## Slide 1
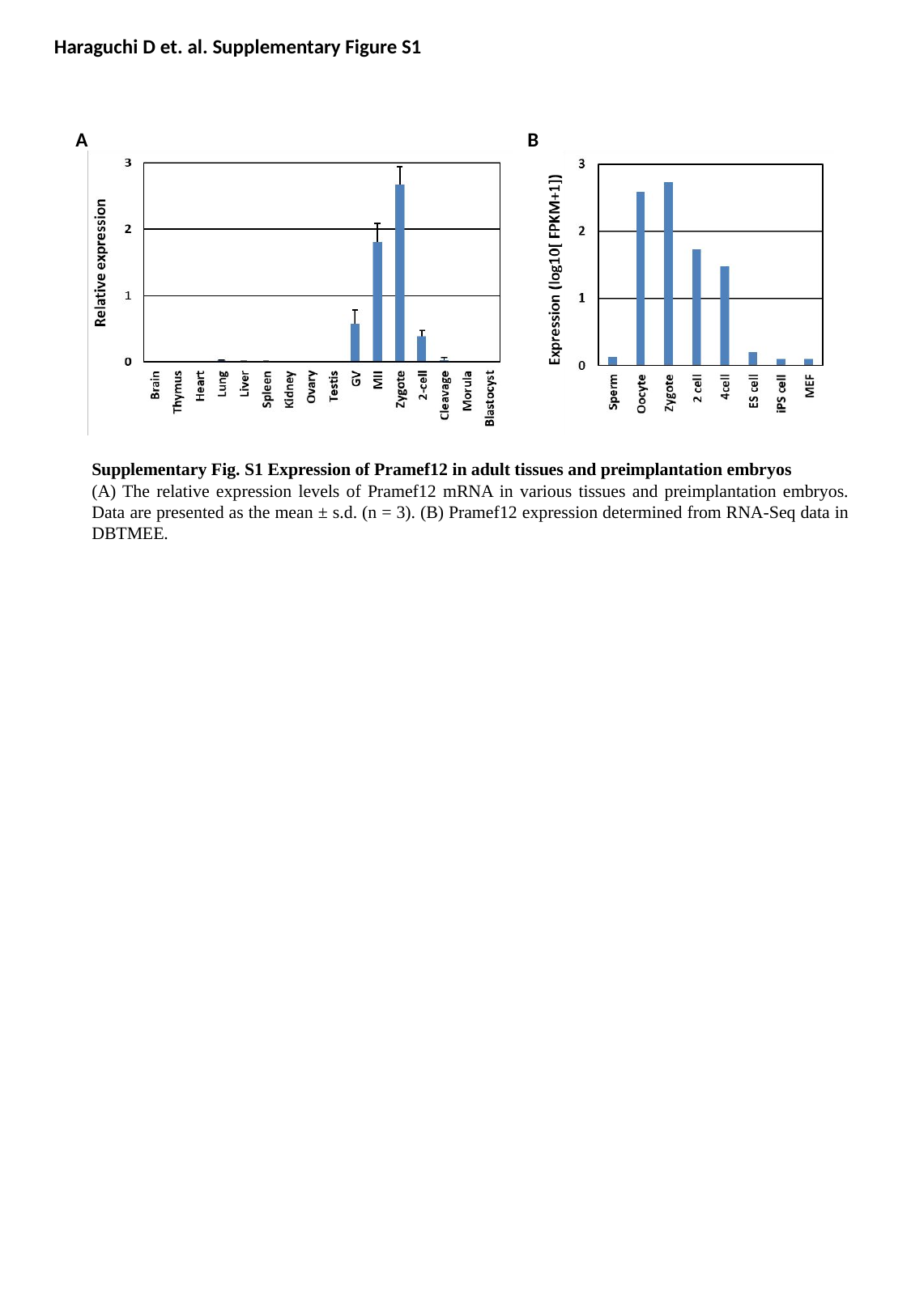

Haraguchi D et. al. Supplementary Figure S1
A
B
Supplementary Fig. S1 Expression of Pramef12 in adult tissues and preimplantation embryos
(A) The relative expression levels of Pramef12 mRNA in various tissues and preimplantation embryos. Data are presented as the mean ± s.d. (n = 3). (B) Pramef12 expression determined from RNA-Seq data in DBTMEE.

## Slide 2
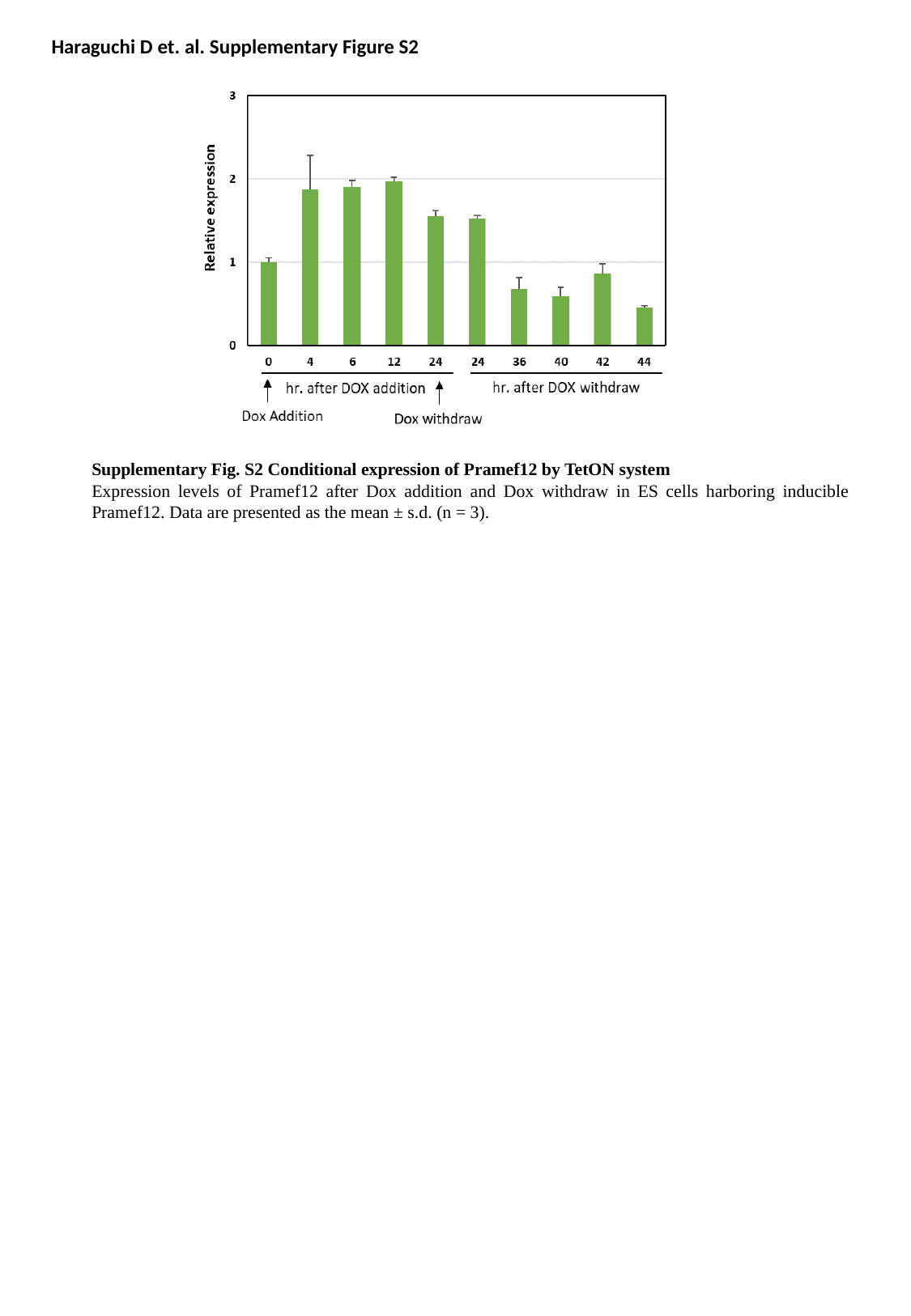

Haraguchi D et. al. Supplementary Figure S2
Supplementary Fig. S2 Conditional expression of Pramef12 by TetON system
Expression levels of Pramef12 after Dox addition and Dox withdraw in ES cells harboring inducible Pramef12. Data are presented as the mean ± s.d. (n = 3).

## Slide 3
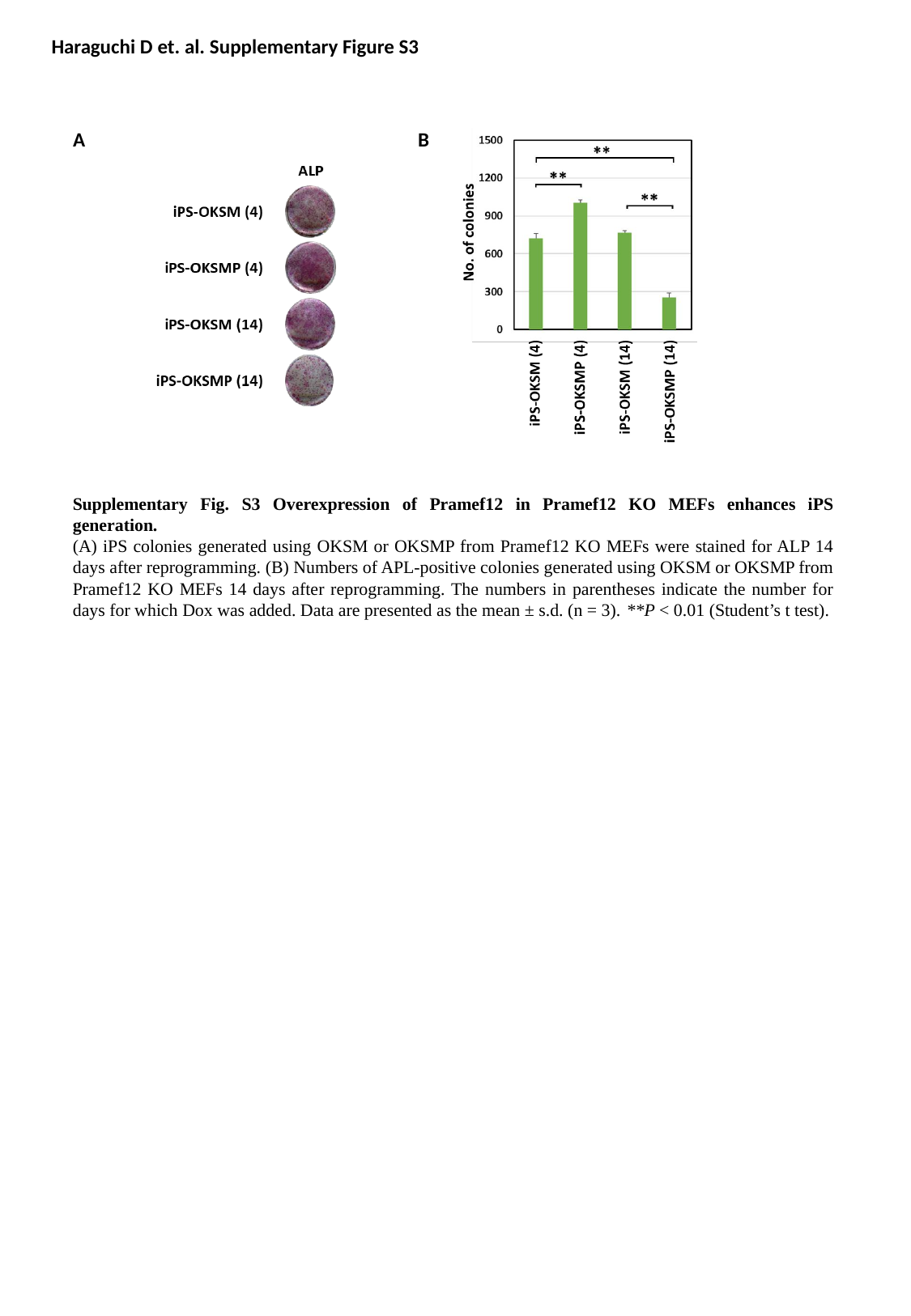

Haraguchi D et. al. Supplementary Figure S3
A
B
Supplementary Fig. S3 Overexpression of Pramef12 in Pramef12 KO MEFs enhances iPS generation.
(A) iPS colonies generated using OKSM or OKSMP from Pramef12 KO MEFs were stained for ALP 14 days after reprogramming. (B) Numbers of APL-positive colonies generated using OKSM or OKSMP from Pramef12 KO MEFs 14 days after reprogramming. The numbers in parentheses indicate the number for days for which Dox was added. Data are presented as the mean ± s.d. (n = 3). **P < 0.01 (Student’s t test).

## Slide 4
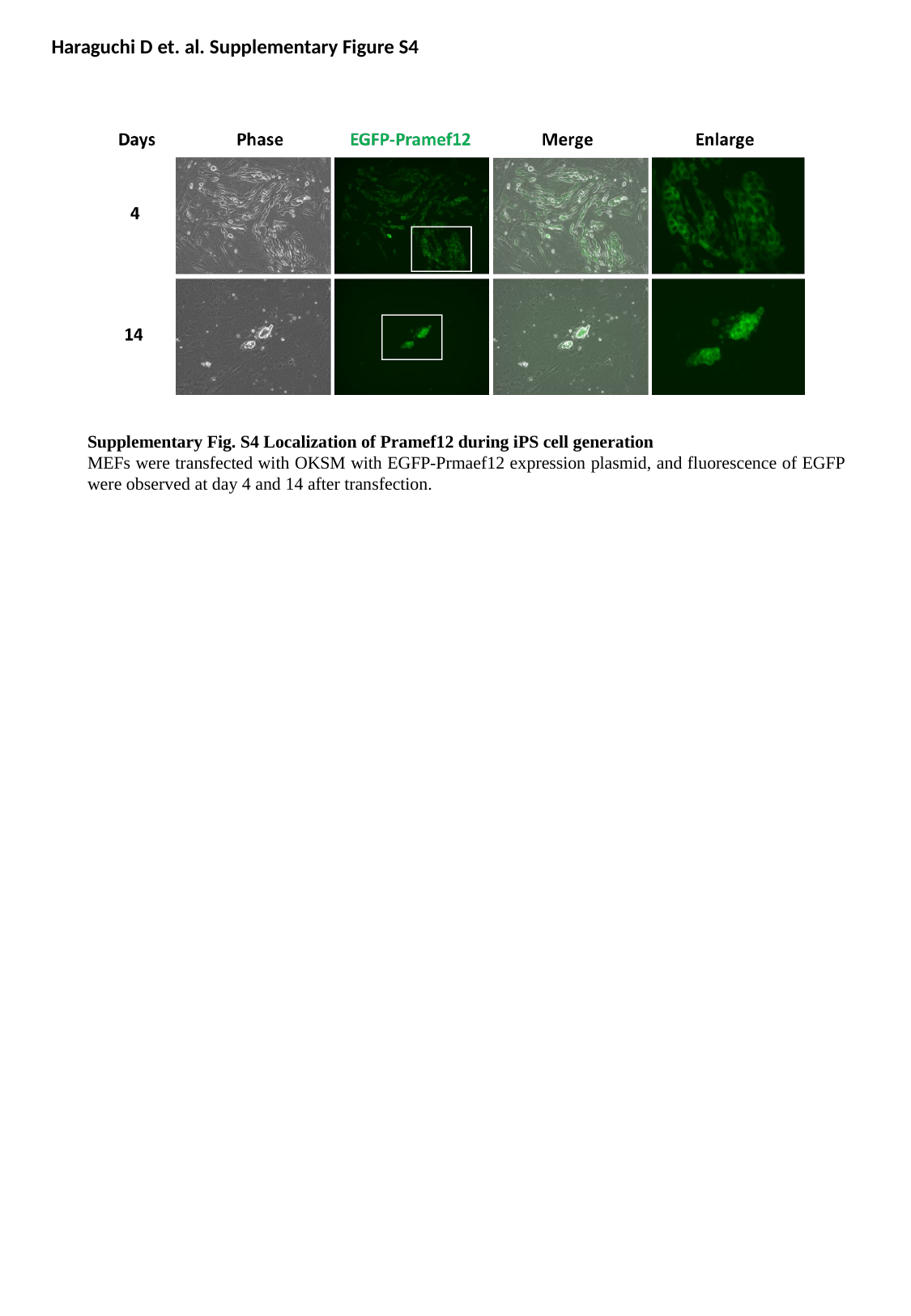

Haraguchi D et. al. Supplementary Figure S4
Supplementary Fig. S4 Localization of Pramef12 during iPS cell generation
MEFs were transfected with OKSM with EGFP-Prmaef12 expression plasmid, and fluorescence of EGFP were observed at day 4 and 14 after transfection.
